# SiC Fin-Shaped Gate Trench MOSFET with Integrated Schottky Diode

**DOI:** 10.3390/ma14227096

**Published:** 2021-11-22

**Authors:** Xiaochuan Deng, Rui Liu, Songjun Li, Ling Li, Hao Wu, Xuan Li

**Affiliations:** 1 State Key Laboratory of Advanced Power Transmission Technology, Global Energy Interconnection Research Institute, Beijing 102209, China; ruilnju@163.com (R.L.); ling_li10@163.com (L.L.); jerrywhtc@163.com (H.W.); 2 School of Electronic Science and Engineering, University of Electronic Science and Technology of China, Chengdu 610054, China; lisongjun@std.uestc.edu.cn (S.L.); xuanli@uestc.edu.cn (X.L.)

**Keywords:** silicon carbide (SiC) trench MOSFET, split P shield (SPS), fin-shaped gate, integrated SBD, transient extreme stress

## Abstract

A silicon carbide (SiC) trench MOSFET featuring fin-shaped gate and integrated Schottky barrier diode under split P type shield (SPS) protection (FS-TMOS) is proposed by finite element modeling. The physical mechanism of FS-TMOS is studied comprehensively in terms of fundamental (blocking, conduction, and dynamic) performance and transient extreme stress reliability. The fin-shaped gate on the sidewall of the trench and integrated Schottky diode at the bottom of trench aim to the reduction of gate charge and improvement on the third quadrant performance, respectively. The SPS region is fully utilized to suppress excessive electric field both at trench oxide and Schottky contact when OFF-state. Compared with conventional trench MOSFET (C-TMOS), the gate charge, Miller charge, *V*_on_ at third quadrant, *R*_on,sp_·*Q*_gd_, and *R*_on,sp_·*Q*_g_ of FS-TMOS are significantly reduced by 34%, 20%, 65%, 0.1%, and 14%, respectively. Furthermore, short-circuit and avalanche capabilities are discussed, verifying the FS-TMOS is more robust than C-TMOS. It suggests that the proposed FS-TMOS is a promising candidate for next-generation high efficiency and high-power density applications.

## 1. Introduction

Silicon carbide (SiC) is an emerging material for power semiconductors with both competitive electric and thermal advantages. This enables SiC central to medium-high voltage power device technology area, where SiC based metal oxide semiconductor field effect transistor (MOSFET) is considered to be the next-generation prime switching device candidate for various applications involving uninterruptible power supply (UPS), photovoltaic (PV) inverter, electric vehicle, etc. [1,2,3,4,5].

SiC MOSFETs still have not reached their expected performance due to low channel mobility. The introduced trench gate can achieve lower specific on-resistance *R*_on,sp_ by means of increasing channel density. On the other hand, the trench gate spells excessive electric field around the bottom and corner of the trench gate, which concerns long-term reliability. Moreover, the trench gate also brings considerable switching loss, which restricts the dynamic advantage of SiC MOSFET [6]. In order to solve these issues, several solutions are proposed at device level: (1) whole P+ shield region implanted at the bottom of trench [7]; (2) P+ shield region under the recessed source region (double trench MOSFET) [8], (3) buried P+ region in the drift region of trench MOSFET [9], (4) deep P base region using ultra-high implantation energy [10], (5) P+ shield region under the part of trench bottom (asymmetric trench MOSFET) [11], and (6) ground/floating split P+ shield region under the bottom of trench [12]. The fin-shape is introduced to reduce the switching loss directly [13]. Furthermore, except for forward conduction, the excellent reverse conduction (i.e., the third quadrant performance) of SiC MOSFET is also desirable for next-generation compact power electronics. From a device design perspective, thus, the Schottky barrier diode (SBD) integrated in SiC MOSFET was an efficient way to avoid bipolar degradation if the parasitic P-N body diode were opened. Specifically, there are several schemes to fulfill: (1) SiC MOSFET with integrated JBS using a same metal scheme (JBSFET) [14,15], (2) various SiC planar/trench gate MOSFETs with integrated SBD between splitting P base region [16,17,18,19,20,21], and (3) SiC trench MOSFET with integrated SBD at sidewall of trench [22].

In this paper, a SiC fin-shaped gate trench MOSFET with integrated Schottky diode (FS-TMOS) is proposed, and its physical mechanism is investigated in terms of static and dynamic performance with TCAD Sentaurus. Furthermore, the transient extreme stress is also considered, involving short-circuit and avalanche capabilities.

## 2. Structure and Mechanism

**Figure 1:**
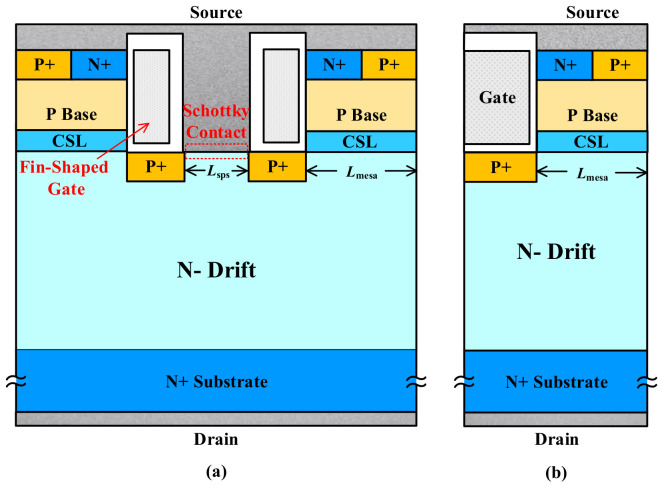
Cross-section view of (**a**) FS-TMOS (cell pitch) and (**b**) C-TMOS (half-cell pitch).

Sentaurus-2018 Technology Computer-aided Design (TCAD) simulators from American Synopsys Inc. are applied to investigate the electrical characteristics of the devices. The cross-section views of FS-TMOS and conventional trench MOSFET (C-TMOS) are illustrated in Figure 1a,b, respectively. Compared with the C-TMOS, there are two proposed structure components: (1) fin-shape and (2) integrated Schottky contact. The fin-shaped gate located on the sidewall of trench is utilized to reduce Miller charge, and the Schottky contact introduced in the trench bottom region between adjacent fin-shaped gate aims to improve the third quadrant performance avoiding bipolar degradation from the P- base region/N drift region formed junction operation. Moreover, the split P shield (SPS) located on the two sides of the trench bottom is introduced under the fin-shaped gate. The SPS in the FS-TMOS facilitates two main functions: (1) One is to protect trench gate oxide from excessive electric field and (2) another is to suppress the electric field of Schottky contact interface, which are of significance to long-term reliability in the OFF-state. No matter the SPS and the conventional P shield, the introduced JFET effect severely degenerates *R*_on,sp_. Hence, the current spreading layer (CSL) is adopted to solve the aforementioned contradiction.

To have a fair comparison between the two structures, the doping concentration and dimension of the fundamental structure are kept the same, except the trench width (FS-TMOS: 2 μm for the merged SBD and C-TMOS: 1 μm) and the gate shape (the width of one fin-shaped gate is 0.5 μm). The thicknesses of Pshield, Pbase, and CSL are 0.2, 0.5, and 0.4 μm, respectively. The two structures are based on an N type drift layer with thickness of 11 μm and doping concentration of 8.0 × 10^15^ cm^−3^. The doping of CSL is 5.0 × 10^16^ cm^−3^. The depth of trench is 1 μm, and the thicknesses of silicon dioxide (SiO_2_) along the sidewall and bottom of trench are 50 and 100 nm, respectively. The gate channel length is 0.5 μm and the nickel (Ni) with a work function of 5.1 eV [1] is adopted for Schottky contact. The SPS and conventional P+ shield region well short-connected with source contact is to alleviate the charge storage effect resulting in dynamic *R*_on,sp_ degradation [23].

Due to high interface state density located at the SiC–SiO_2_ interface, channel mobility is degraded evidently compared with theoretical mobility. Inverse and Accumulation Mobility Model (IALMob) and Interface Charge model in Enormal are called considering Coulomb impurity scattering and charged traps and fixed charge scattering. Nowadays, the channel mobility (up to 20–50 cm^2^/V·s) for SiC MOSFET can be achieved upon nitridations of the gate oxide [24]. The channel mobility is adjusted to 30 cm^2^/V·s in this work. Moreover, the Shockley Read–Hall recombination Model, Auger Recombination Model, Doping-Dependent Mobility Model, Ionization Mobility Model, High-Field Saturation Model, Incomplete Ionization Model, Inversion and Accumulation Layer Mobility Model, Interface Charge Mobility Degradation Model, Hatakeyama Avalanche Model, Thermodynamic Model, and Thermoelectric Power Model are adopted in this simulation. Considering the anisotropic properties of SiC, the Anisotropic Model of Mobility and Avalanche is used also [25].

## 3. Results and Discussion

**Figure 2:**
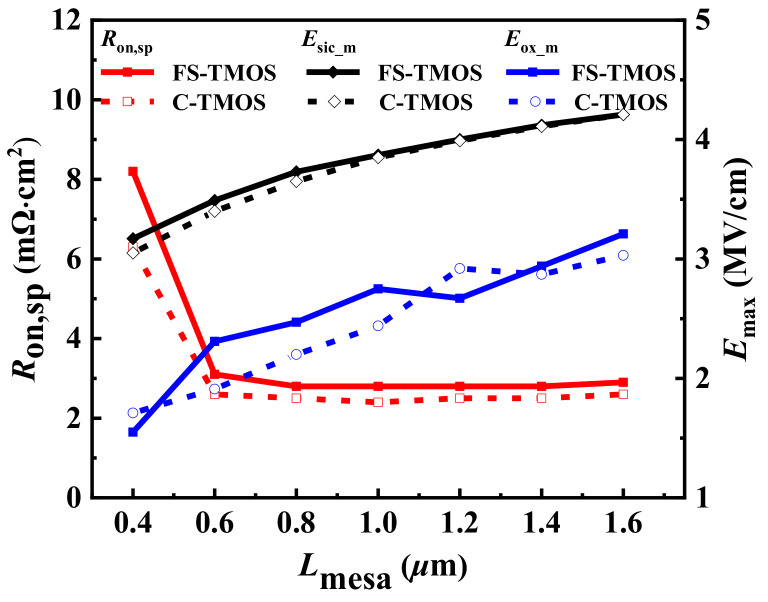
*E*_ox_m_ and *E*_sic_m_ (when the drain-source voltage *V*_ds_ = 1200 V) and *R*_on,sp_ dependent on *L*_mesa_.

**Figure 3:**
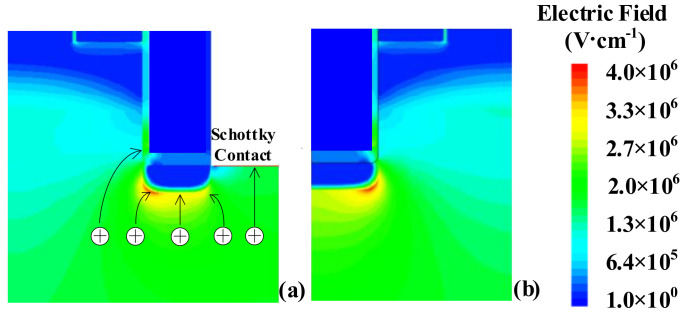
Electric field distribution of FS-TMOS (**a**) and C-TMOS (**b**).

The influence of mesa width *L*_mesa_ on maximum electric field of trench gate SiO_2_ *E*_ox_m_ and SiC *E*_sic_m_ and *R*_on,sp_ is illustrated in Figure 2. Due to relatively lower channel density of the FS-TMOS, the *R*_on,sp_ of FS-TMOS is greater than that of C-TMOS. When the *L*_mesa_ is lower than 0.6 μm, the *R*_on,sp_ of the two TMOS increases evidently as a result of the JFET pinch-off effect. Whereas the *L*_mesa_ is larger than 1.6 μm, the punch-through effect happens at the P Base region. Because the introduced P shield is able to alleviate the electric field crowding effect around trench corner and bottom, the *E*_ox_m_ is expected to be much lower than the long-time reliability concern value 3~4 MV/cm. Apart from screening trench gate oxide from electric field crowding, the SPS plays another important role by absorbing the electric field line, overwhelming the excessive electric field located at the Schottky contact, simultaneously, as shown in Figure 3.

**Figure 4:**
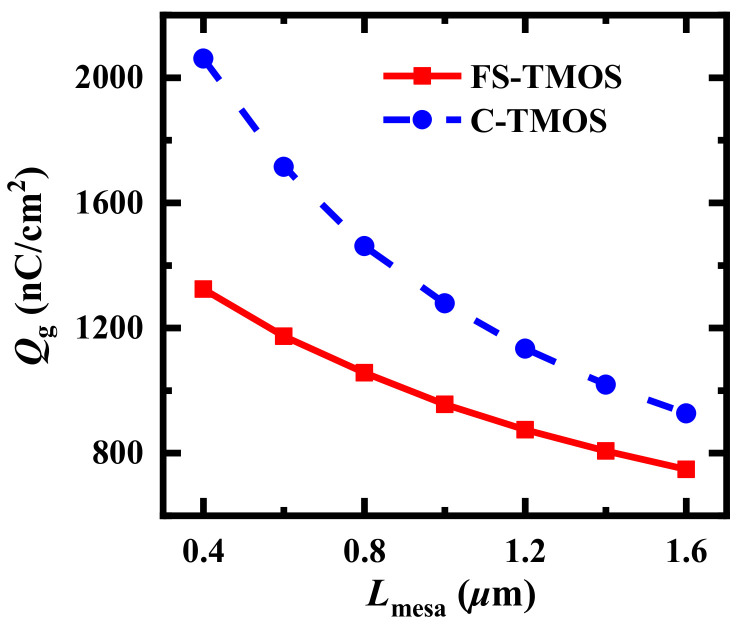
*Q*_g_ of FS- and C-TMOS dependent on *L*_mesa_. The *L*_sps_ is 1*μ*m.

Moreover, the influence of *L*_mesa_ on gate charge (*Q*_g_, which is achieved from the integral of gate current from the gate-source voltage *V*_gs_ = −5 V to *V*_gs_ = +15 V) is illustrated in Figure 4. The *Q*_g_ of FS-TMOS is significantly reduced because the advantage of fin-shaped gate can reduce the effective overlap area between gate and source terminal.

**Figure 5:**
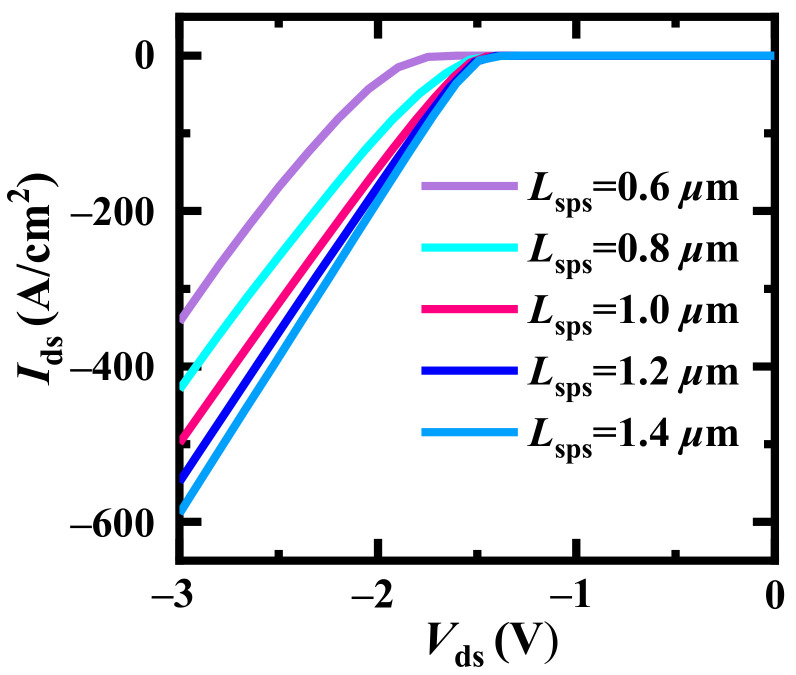
Third quadrant characteristics of FS-TMOS dependent on *L*_sps_.

Furthermore, the distance between the two adjacent SPS *L*_sps_ influences the third quadrant performance as shown in Figure 5. With wider *L*_sps_, the reverse conduction capability (the drain-source current, *I*_ds_) enhances gradually. On the other hand, the turn on voltage (*V*_on_) is degraded evidently when *L*_sps_ is lower than 0.8 μm. Considering state-of-the-art process technologies for SiC power devices and fundamental capability, the *L*_mesa_ and *L*_sps_ are optimized to be fixed 1 μm and 1 μm, respectively, in the following discussions.

**Figure 6:**
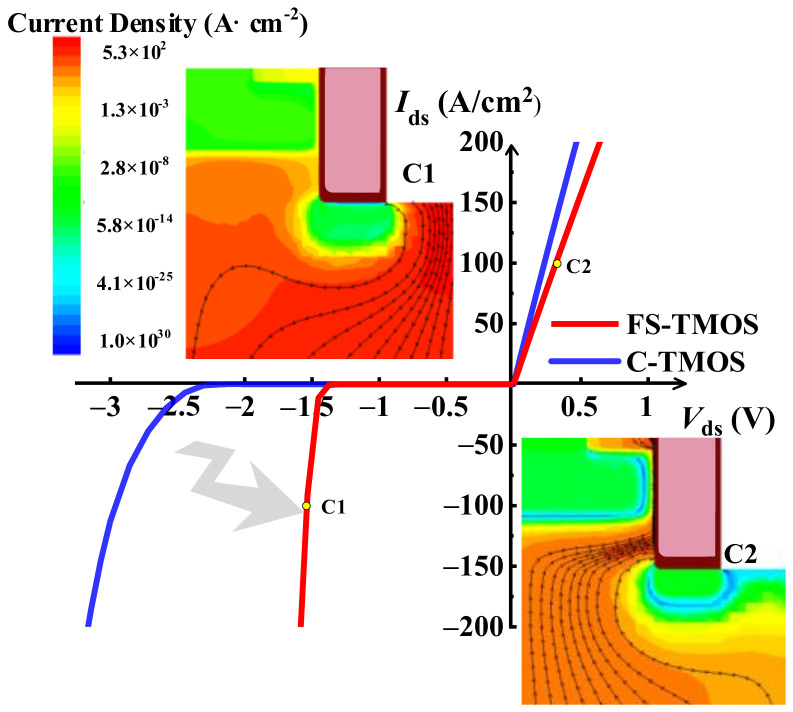
Forward and reverse characteristics of both optimum FS- and C-TMOS. The current distribution at the two key states C1 and C2 is presented as the inner figure.

The distribution of current density and the current flowing path inside the FS-TMOS at forward and reverse conductive state (i.e., C1 and C2 point of Figure 6) are depicted in the insets of Figure 5. The reverse current flowing through Schottky contact when *I*_ds_ is 100 A/cm^2^ indicates the desirable inactivation of P body/N drift formed diode in reverse conduction operation. The *V*_on_ of FS-TMOS is reduced to 1.5 V.

**Figure 7:**
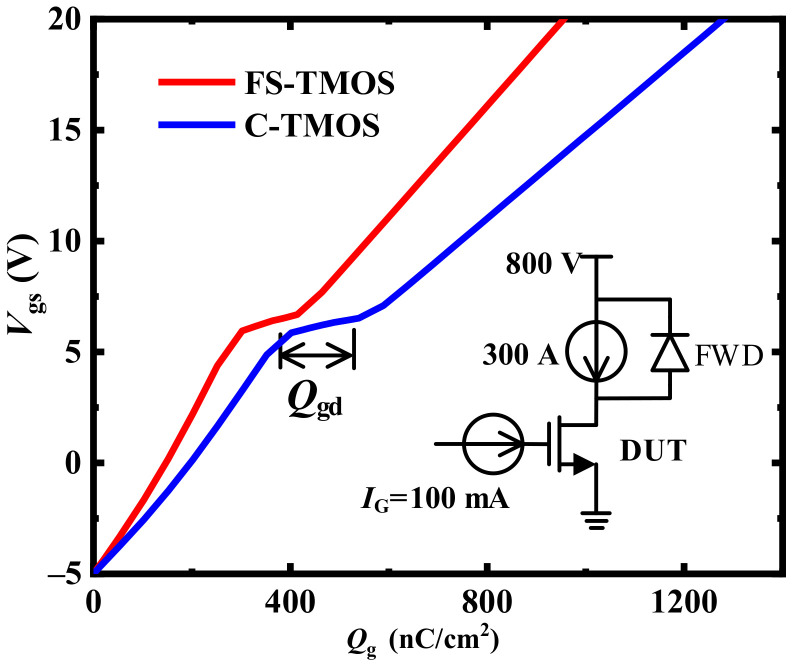
Gate a charge of optimum FS- and C-TMOS.

Furthermore, the relationship between charge stored in C_iss_ (*Q*_g_) and *V*_gs_ of FS- and C-TMOS is illustrated in Figure 7. The *Q*_g_ of FS- and C-TMOS are 964 nC/cm^2^ and 1290 nC/cm^2^, respectively. The gate-to-drain charge (*Q*_gd_) of FS- and C-TMOS are 162 nC/cm^2^ and 194 nC/cm^2^, respectively. It shows that the *Q*_g_ and *Q*_gd_ of FS-TMOS are significantly reduced by 34% and 20%, compared with C-TMOS.

**Figure 8:**
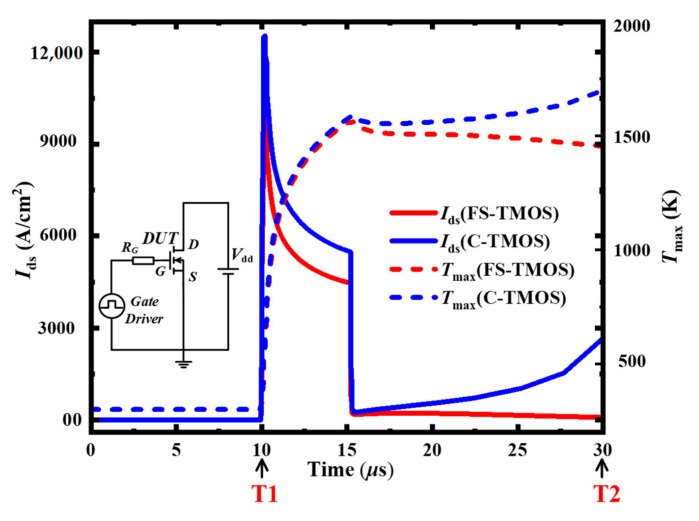
Short-circuit waveforms of FS- and C-TMOS under 600 V DC bus voltage. The *T*_max_ means the maximum temperature inside the FS- and C-TMOS at each time point.

**Figure 9:**
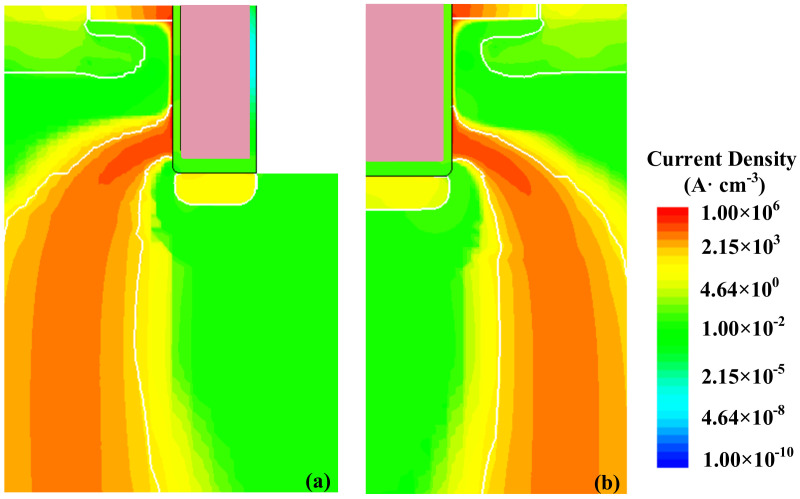
Current distribution inside (**a**) FS- and (**b**) C-TMOS at the time point T1.

Apart from fundamental performance, the transient extreme stress short circuit and avalanche is also discussed in detail. Electrothermal simulations are carried out to estimate the temperature distribution in the following part. According to the actual package situation, the device is thermally an adiabatic boundary, except for the drain electrode, the bottom surface of the device, which is a thermally conducting boundary. The “Source” and “Gate” thermal contacts are considered as thermal insulators, whereas the “Drain” thermal contact serves as the only thermally conducting boundary. The junction-to-case thermal resistance *R*_th,j-c_ = 0.6 K/W. In short-circuit stress under the DC bus voltage 600 V, the waveforms of the FS- and C-TMOS are shown in Figure 8. The FS-TMOS can survive more than 5 μs short-circuit stress (*t*_sc_) while the C-TMOS is down disastrously. The saturation current of FS-TMOS is evidently lower than that of C-TMOS due to lower channel density of FS-TMOS. The maximum temperature during short-circuit shock is estimated approximately 1500 K. References [26,27,28] reported similar simulation results. In the initial state of the short-circuit process (point T1), the current distribution inside FS- and C-TMOS is very similar to the one shown in Figure 9.

**Figure 10:**
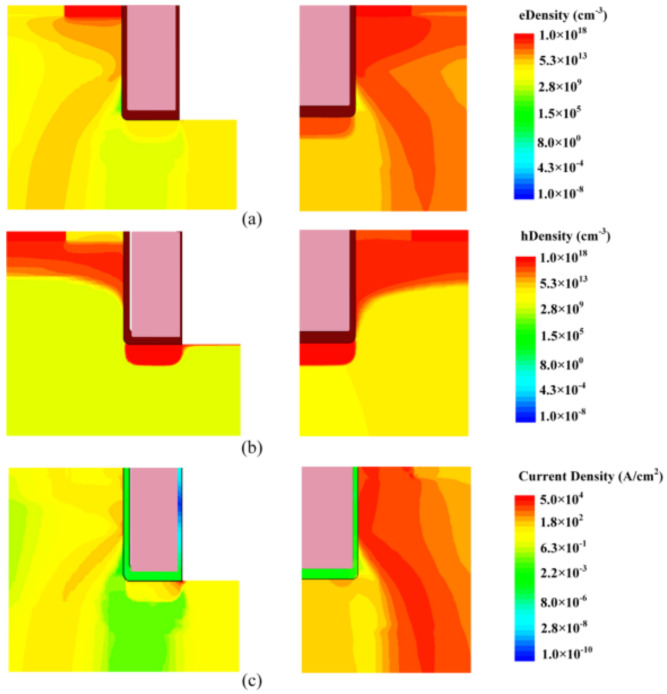
(**a**) Electron, (**b**) hole density distribution, and (**c**) current distribution inside (left) FS- and (right) C-TMOS at the time point T2.

Although the gate channel is turned off after 15 μs, the C-TMOS is out of control afterwards, while the FS-TMOS survives. During this process, the junction temperature is high enough to make minor carrier increase exponentially, which results in a current path between drain and source terminal. If the dissipated power exceeds the power moving away from device, the temperature continues to increase, leading to device failure. The electron and hole distribution and formed current of FS- and C-TMOS at the time point T2 are shown in Figure 10.

**Figure 11:**
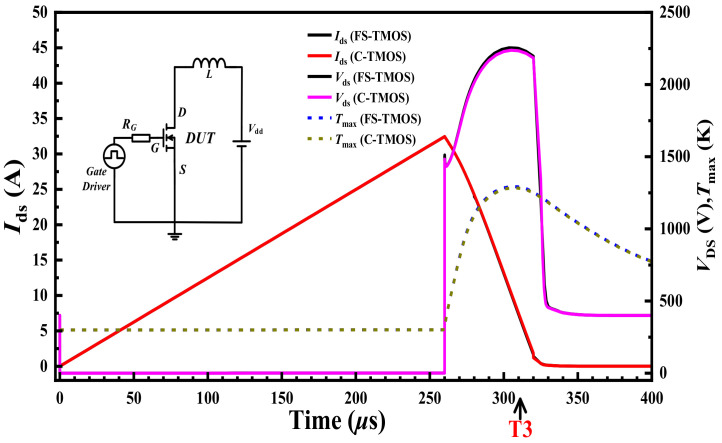
UIS waveforms of FS- and C-TMOS.

Avalanche is another important transient extreme stress shock, when high voltage of the MOSFET exceeds its maximum rating as a result of back-electromotive force from L, although the MOSFET is turned off. The avalanche capabilities of FS- and C-TMOS are shown in Figure 11 by unclamped inductive switching UIS circuit.

**Figure 12:**
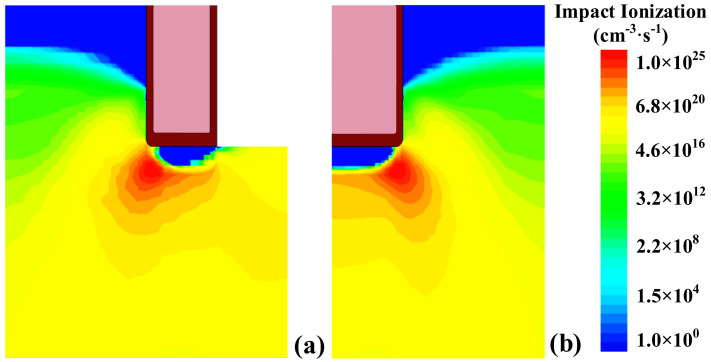
Impact ionization distribution inside (**a**) FS- and (**b**) C-TMOS at the time point T3.

Although an SBD is merged in the fin-shape trench gate, the maximum avalanche energy (*E*_av_) and the maximum temperature during this process are almost the same for the FS- and C-TMOS. The impact ionization distributions of FS- and C-TMOS at the time point T3 are also verified by the results, as shown in Figure 12. In other words, the integrated Schottky diode between the FS gate does not degrade the avalanche capability. It is suggested that the avalanche failure of the two devices tends to be the same.

The key parameters of the two devices are summarized in Table 1. The unipolar device-level Figure of Merit [29] *R*_on,sp_·*Q*_gd_ and *R*_on,sp_·*Q*_g_ of FS-TMOS are almost the same and improved by 14% more than those of C-TMOS, respectively, validating advantage of comprehensive performance of FS-TMOS.

## 4. Conclusions

The FS-TMOS is proposed featuring FS trench gate, integrated Schottky diode between the gates, and SPS structure, in this paper. The corresponding physical mechanism is studied in terms of fundamental (blocking, conduction, and dynamic) performance and transient extreme stress reliability. The SPS is tactfully introduced to shield trench gate oxide and Schottky contact at trench bottom simultaneously. Compared with C-TMOS, the *Q*_g_, *Q*_gd_, *V*_on_, *R*_on,sp_·*Q*_gd_, and *R*_on,sp_·*Q*_g_ of FS-TMOS are reduced by 34%, 20%, 65%, 0.1%, and 14%, respectively, except degradation of *R*_on,sp_. The *t*_sc_ of FS-TMOS is better than C-TMOS and avalanche capability of the two devices is very similar. It verifies FS-TMOS is a next-generation SiC MOSFET with competitive performance for efficient and reliable high frequency applications.

## Figures and Tables

**Table 1:** Characteristics of FS- and C-TMOS.

Parameter	FS-TMOS (*L*_mesa_ = 1.0 and *L*_sps_ = 1.0)	C-TMOS (*L*_mesa_ = 1.0)	Unit (μm)
*R*_on,sp_	2.8	2.4	mΩ·cm^2^
*Q*_gd_	162	194	nC/cm^2^
*Q*_g_	964	1290	nC/cm^2^
*V*_on_	1.80	2.98	V
*t*_sc_	>5	<5	μs
*E*_av_	>22.8	>22.8	J/cm^2^
*R*_on,sp_·*Q*_gd_	460	464	mΩ·nC
*R*_on,sp_·*Q*_g_	2737	3109	mΩ·nC

## Data Availability

The data presented in this study are available on request from the corresponding author.
